# L-Canavanine, a Root Exudate From Hairy Vetch (*Vicia villosa*) Drastically Affecting the Soil Microbial Community and Metabolite Pathways

**DOI:** 10.3389/fmicb.2021.701796

**Published:** 2021-09-27

**Authors:** Hossein Mardani-Korrani, Masaru Nakayasu, Shinichi Yamazaki, Yuichi Aoki, Rumi Kaida, Takashi Motobayashi, Masaru Kobayashi, Naoko Ohkama-Ohtsu, Yosei Oikawa, Akifumi Sugiyama, Yoshiharu Fujii

**Affiliations:** ^1^Institute of Agriculture, Tokyo University of Agriculture and Technology, Fuchu, Japan; ^2^Research Institute for Sustainable Humanosphere, Kyoto University, Uji, Japan; ^3^Tohoku Medical Megabank Organization, Tohoku University, Sendai, Japan; ^4^Graduate School of Agriculture, Kyoto University, Kyoto, Japan

**Keywords:** allelopathy, hairy vetch (*Vicia villosa* roth), L-canavanine, soil microbial community (SMC), soil metabolites pathways, cover crop

## Abstract

L-Canavanine, a conditionally essential non-proteinogenic amino acid analog to L-arginine, plays important roles in cell division, wound healing, immune function, the release of hormones, and a precursor for the synthesis of nitric oxide (NO). In this report, we found that the L-canavanine is released into the soil from the roots of hairy vetch (*Vicia villosa*) and declines several weeks after growth, while it was absent in bulk proxy. Hairy vetch root was able to exudate L-canavanine in both pots and *in vitro* conditions in an agar-based medium. The content of the L-canavanine in pots and agar conditions was higher than the field condition. It was also observed that the addition of L-canavanine significantly altered the microbial community composition and diversity in soil. Firmicutes and Actinobacteria became more abundant in the soil after the application of L-canavanine. In contrast, Proteobacteria and Acidobacteria populations were decreased by higher L-canavanine concentration (500 nmol/g soil). Prediction of the soil metabolic pathways using PICRUSt2 estimated that the L-arginine degradation pathway was enriched 1.3-fold when L-canavanine was added to the soil. Results indicated that carbon metabolism-related pathways were altered and the degradation of nitrogen-rich compounds (i.e., amino acids) enriched. The findings of this research showed that secretion of the allelochemical L-canavanine from the root of hairy vetch may alter the soil microbial community and soil metabolite pathways to increase the survival chance of hairy vetch seedlings. This is the first report that L-canavanine acts as an allelochemical that affects the biodiversity of soil microbial community.

## Introduction

Amino acids as a significant portion of the root exudates play key roles in shaping the rhizosphere microbial community structure (Hu et al., 2018; Canarini et al., 2019). Rhizosphere amino acids often result from lysis and cellular efflux from plants and soil organisms or proteolysis of existing peptides. They primarily serve as carbon and nitrogen sources in the rhizosphere (Moe, 2013). Amino acids in the soil are an important microbial pool factor, affecting the physiological function of the soil microbial communities (SMC) and can resonate with global scale nutrient biogeochemistry. Although few studies have focused on the effect of the proteinogenic amino acids on the total SMC, the role of many non-proteinogenic free amino acids (FAAs) produced by plants including L-canavanine from hairy vetch is largely unknown (Aliashkevich et al., 2018).

Few amino acids have been isolated and identified as intermediate compounds of biological phenomena such as allelopathy (Araya et al., 2014). It may be because evaluating FAAs is challenging, since the concentration of FAAs available in plant roots and rhizosphere depends on many factors such as soil type (Cao et al., 2016), soil pH (Rothstein, 2010), temperature, elevation, and analytical methods (Young et al., 1974; Chen et al., 2015). For instance, in many cases FAAs are lost by leaching the soil solution (Raab et al., 1996) or during the analytical procedures.

L-Isomeric amino acids and oligopeptides are the key nitrogen source for plants and have a significant effect on the soil organisms’ dynamics, including bacteria and fungi (Jämtgård, 2010; Broughton et al., 2015). So far, there is neither any evidence that the hairy vetch root releases L-canavanine or similar non-proteinogenic amino acids, nor its effect on the soil organism is known. Understanding the hairy vetch root exudates components (i.e., L-canavanine) and their mechanism of action on the soil in organic farming practices is important due to the emerging food security issues caused by climate change.

In plants, seeds often contain a diverse range of compounds during germination that may impact the population and behavior of the soil microorganism in the soil or around the seed environment (Nelson, 2018).

It is well-known that plants often utilize a specific phenomenon called “allelopathy” to combat the competition and/or control their surrounding environment by releasing chemical(s) known as allelochemical (Rice, 2012). Allelochemical can be classified into different groups, including alkaloids, phenols, amino acids, peptides (Fujii and Hiradate, 2007; Rice, 2012), terpenes, ketones, aldehydes (Mardani et al., 2015, 2019), and water-soluble organic acids (Syed et al., 2014).

Also, certain amino acids act as allelochemical or are involved in chemotaxis (Fujii and Hiradate, 2007; Araya et al., 2014). Chemotaxis is a phenomenon by which the chemicals released from seeds stimulate the growth and trivial of the beneficial bacteria toward germinating seeds (Harborne et al., 1971; Peters et al., 1986; Barbour et al., 1991). The current and past trends in chemotaxis research in allelopathy have primarily focused on the major of compounds released from the seeds, including flavonoids, carbohydrates, etc. (Peters et al., 1986; Hartwig et al., 1991). However, the influence of seedling-released amino acids, specifically non-proteinogenic amino acids, on the SMC in organic cultivation practices is largely unexplored. Plants are a great source of proteinogenic or non-proteinogenic amino acids. The presence of non-proteinogenic amino acids is very common in the plant kingdom. In the Leguminosae family alone, over 60 non-proteinogenic amino acids have been isolated and identified (Bell et al., 1977). Plants use these amino acids to interact with their surrounding environments. Several non-proteinogenic amino acids have been identified to protect plant seeds against insects. For example, L-3,4-dihydroxyphenylalanine (L-dopa) and 5-hydroxy-L-tryptophan are available in high concentrations in *Mucuna pruriens* and *Griffonia simplicifolia* and protect the seeds of these species against insect damage (Harborne et al., 1971; Bell et al., 1977; Fujii et al., 1992), although, L-dopa has long been known for its allelopathic activity against other plants (Fujii et al., 1992). Moreover, some of these amino acids are known to be toxic to other plants and other organisms (Nakajima et al., 2001), or act as nitrogen source for other plants and microbes.

Hairy vetch (*Vicia villosa* Roth subsp. *villosa*) is used as a cover crop or a green manure crop in Japanese orchards and rice fields due to several beneficial characteristics, from weed control to suppression of bacterial and fungal diseases (Fujii, 2003). Hairy vetch can control weeds by releasing cyanamide in the form of a volatile compound (Kamo et al., 2003). Kamo et al. later showed that the cyanamide is biosynthesized from amino acid L-canavanine, a non-proteinogenic amino acid present abundantly in hairy vetch seed and sprouts. L-Canavanine has been reported as one of the main allelochemicals of jack bean with plant inhibitory activity (Nakajima et al., 2001). Nakajima et al. (2001) also found that the suppression of the arginine metabolism in the plant is the main mechanism of the allelopathic activity of L-canavanine. Sasamoto et al. (2019) recently showed that the epicotyl protoplasts of hairy vetch contain high level of canavanine. We have also found that L-canavanine at 10 μM suppressed the division of lettuce protoplasts, which was more severe than that of cyanamide (Sasamoto et al., 2019).

In this study, we aimed to know if the L-canavanine in the hairy vetch seeds could be released by seedling roots and influence the soil microbial community in the early stages of plant establishment. The findings would provide a better understanding of the reason for the accumulation of non-proteinogenic amino acid in the seeds and their role in plant interaction with microorganisms.

## Results

### L-Canavanine Exudation From the Hairy Vetch Roots

**FIGURE 1:**
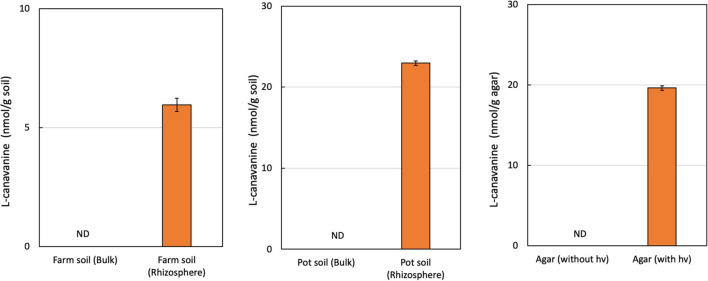
L-Canavanine concentration under several experimental conditions. The content of the L-canavanine was measured in the Bulk and Rhizosphere soil of hairy vetch in the farm condition along with Pot and Agar medium conditions. ND, not detected; each bar presents the mean ± SD of three biological replicates.

We extracted FAAs from bulk soil and the rhizosphere of hairy vetch seedlings. Using GC-MS analysis, we detected an L-canavanine derivative only in the rhizosphere soil of both farm soil (8 nmol/g soil) and greenhouse (23 nmol/g soil), but not in the bulk soil. Our results indicated that L-canavanine is secreted from the hairy vetch roots into the rhizosphere soil in considerable amounts (Figure 1). We estimated the L-canavanine concentration in the farm and greenhouse condition (pots) to evaluate the exudation in different environments.

In the next step, by growing hairy vetch on agar medium, we validated that L-canavanine can be exudated by the hairy vetch root into the surrounding substrate.

**FIGURE 2:**
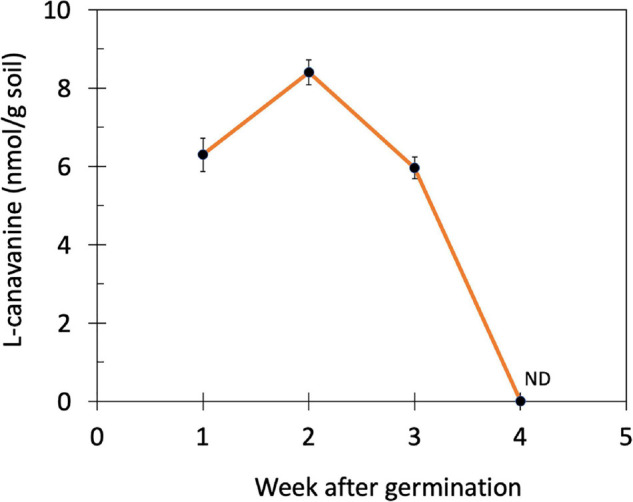
Changes in the L-canavanine content of the rhizosphere soil of hairy vetch over 4 weeks after germination (week 1 to week 4) in the farm condition. ND, not detected; Error bars present the mean ± SD of three biological replicates.

Subsequently, the changes in the L-canavanine content in the rhizosphere soil of hairy vetch were monitored over 4 weeks after germination and during the vegetative stage when plants were 7 cm (week 1), 10 cm (week 2), 15 cm (week 3), and 20 cm (week 4) height (Figure 2). The release of L-canavanine continued to increase in the rhizosphere as the seedling grew up to the 2nd week. After that, it started declining to an undetectable level on the 4th week (Figure 2).

### The Effects of the L-Canavanine on the Soil Microbial Community Dynamics

We then examined the effect of L-canavanine concentrations (low, medium, and high) on SMC and predicted functional potentials of microbes by the 16S rRNA amplicon sequencing analysis. The microbiome analysis in this study showed that the addition of the L-canavanine in 0 (control), 20 (low), 100 (medium), and 500 (high) nmol/g soil concentrations changed the SMC. In comparison with the control, this effect of L-canavanine was more pronounced at high concentration (500 nmol/g soil). Although this concentration was high in the farm and pot soil, in this study, the microbiome analysis was only done for the incubation test and not for the rhizosphere soil of hairy vetch.

**FIGURE 3:**
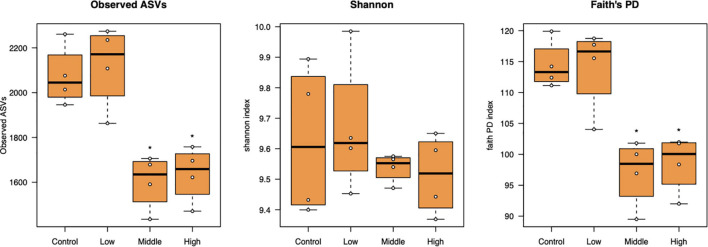
Alpha diversity of the bacterial community in soil treated with low (20 nmol/g soil), medium (100 nmol/g soil), and high (500 nmol/g soil) concentrations of L-canavanine in comparison with the control (0 nmol) (*FDR < 0.05, significant difference from the control, pairwise Kruskal–Wallis test corrected by the Benjamini–Hochberg method).

Bacterial alpha diversity as represented by the number of observed ASVs and Faith’s phylogenetic diversity significantly decreased at the medium to high L-canavanine concentrations in soil, but the Shannon index did not show any significant changes (Figure 3).

**FIGURE 4:**
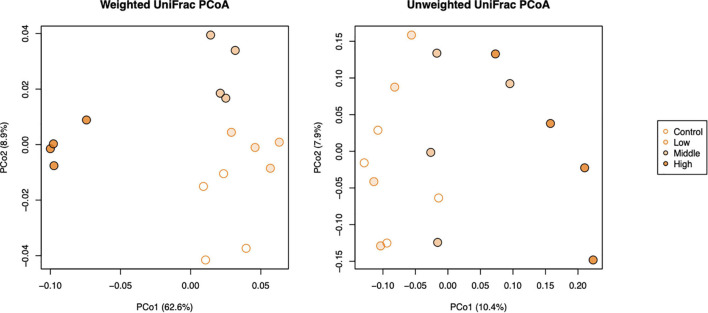
Principal coordinate analysis of weighted and unweighted UniFrac distances of soil bacterial communities (beta diversity) after treatment with L-canavanine in comparison with control.

The beta diversity of bacterial communities in different applied concentrations as displayed by principal coordinate analysis (PCoA) of weighted and unweighted UniFrac (WUF and UUF) distances revealed that L-canavanine had significant impact on microbiome community structure. Both WUF and UUF PCoA ordination plots showed that the community structure at the high concentration of L-canavanine was distinct from those at none to medium concentration on axis one (Figure 4). The permutational multivariate analysis of variance (PERMANOVA) based on WUF and UUF distances confirmed that community structures were significantly different among concentrations (WUF, *R*^2^ = 0.711, *p* < 0.001; UUF, *R*^2^ = 0.229, *p* = 0.002), and the WUF and UUF distances from the control were increased by L-canavanine addition in a dose-dependent manner (Supplementary Figure 1).

**FIGURE 5:**
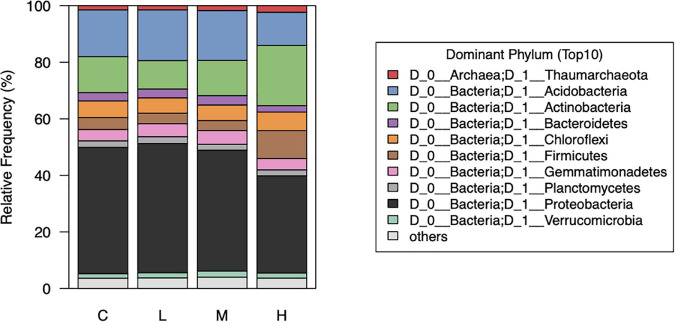
Relative abundance of the top 10 dominant soil bacterial taxa at phylum level after treatment with low, medium, high, and control (0) concentrations of L-canavanine.

We noticed that the abundance of bacteria at the phylum level were notably different between the control (0 nmol/g soil) and high (500 nmol/g soil) concentration of L-canavanine (Figure 5). The relative abundance of Acidobacteria and Proteobacteria was low and Firmicutes and Actinobacteria were high at the high L-canavanine level.

**FIGURE 6:**
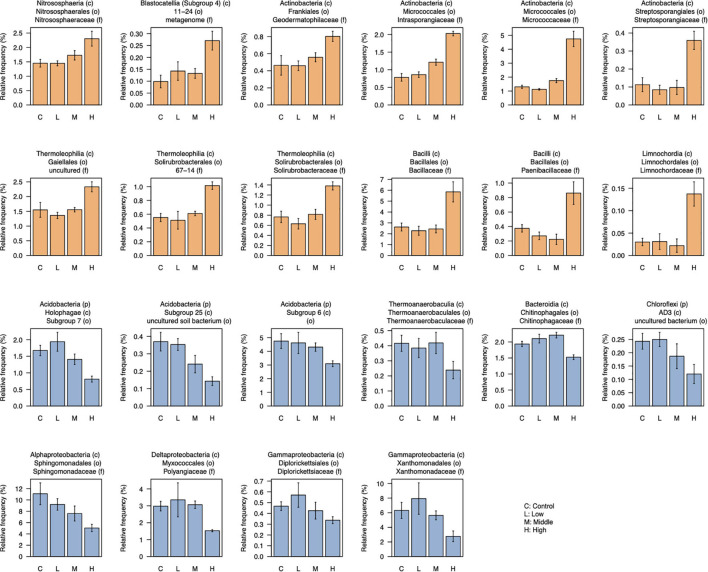
Relative abundance of taxa (family level) that were differentially abundant between control and high concentration of L-canavanine. The 12 taxa were significantly enriched (orange bar), and 10 taxa were significantly depleted (blue bar) after treatment with L-canavanine (FDR < 0.05, Welch’s *t*-test corrected by the Benjamini–Hochberg method).

After filtering low-abundant taxa (mean < 0.05%), 176 taxa at the family level were used for the differential abundant test between control and high L-canavanine treatment. The abundance of certain families differed and was more distinct between control and high concentration of L-canavanine. In particular, 12 taxa were enriched, while 10 others were depleted significantly at high L-canavanine treatment compared with the control (Figure 6). Of these, some taxa such as Micrococcaceae and Intrasporangiaceae, showed concentration-dependent enrichment, and some taxa in Acidobacteria and Sphingomonadaceae showed concentration-dependent depletion. In addition, at the family level, Bacillaceae and Micrococcaceae families were most dominant after application of L-canavanine, and their abundance increased significantly (Figure 6).

### Prediction of Metagenome Functions From 16S Amplicon Data

**FIGURE 7:**
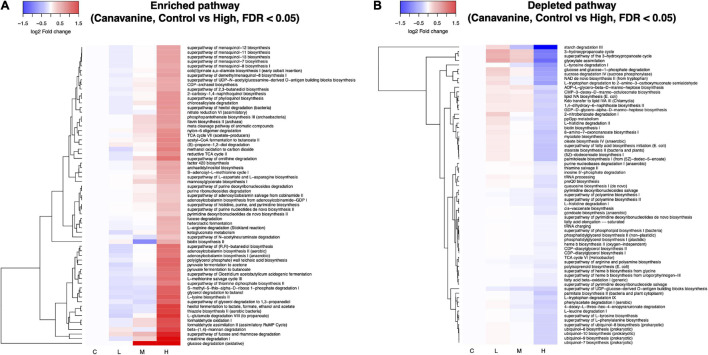
Heatmap depiction of inferred microbial metabolic pathways that were differentially abundant between control and high concentration of L-canavanine. The functional prediction analysis with PICRUSt2 revealed that the soil treatment with L-canavanine significantly enriched **(A)** 65 and depleted 67 **(B)** metabolic pathways, respectively. Differential abundance of the pathway between the control and high concentration of substrate L-canavanine was tested using ALDEx2 with FDR < 0.05 cutoff.

We used PICRUSt2 to predict the metagenome functions based on the 16S amplicon sequences (Douglas et al., 2020). Soil treatment with L-canavanine caused extensive changes in the function of the soil metabolic pathways. A total of 439 pathways were predicted, and differential abundant test was performed between control (0 nmol, not affected) and high concentrations of L-canavanine (500 nmol/g soil, most affected) using the ANOVA-like differential expression (ALDEx2) analysis (Fernandes et al., 2013, 2014). ALDEx2 analysis indicated that 65 pathways were significantly enriched while 67 pathways were significantly depleted after soil inoculation with L-canavanine (Figures 7A,B and Supplementary Figure 2). The heatmap of the fold changes of the pathways affected by L-canavanine indicated that most affected metabolic pathways related to compounds used or produced in the carbon cycle such as “starch degradation III,” “glyoxylate assimilation” (a process that allows bacteria to produce 3-hydroxypropionate utilizing in carbon dioxide) were depleted (Figure 7B). Our observation showed that “glucose degradation (oxidative)” pathway along with the major metabolic pathways related to compounds used or produced during the degradation of amino acids including “creatine degradation I,” “L-glutamate degradation VII (to propanoate),” “superpathway of ornithine degradation,” and “L-arginine degradation (Stickland reaction)” was significantly enriched when L-canavanine was added to the soil (Figure 7A).

## Discussion

Amino acids are released from the plant roots and serve numerous purposes. The exudation of proteinogenic FAAs from plants affects SMC and characteristics (Moe, 2013). They are released in different types and quantities depending on the plant species and environmental factors (Cao et al., 2016). Studies on the concentration of the FAAs in the soil have been reported (Raab et al., 1996, 1999; Fernández-Aparicio et al., 2017).

To examine the role of root exudated amino acids on soil nitrogen budget and microbiome, the influence of numerous factors such as the availability and ease of uptake by the plant root system needs to be considered (Schulten and Schnitzer, 1997). Thus, in this study we only focused on the exudation of L-canavanine from hairy vetch root. The observed differences between the pot and field conditions at the same vegetative stages could be due to the difference in the soil parameters, temperature, and environmental condition (Dennis et al., 2019). Also, seasonal precipitation in field soil could be a reason for lower detection of L-canavanine. We sampled the field soil when temperature fluctuates over the months and might affect the biological activity in the soil. Similar to our observation regarding the decline in L-canavanine in the soil, diminution in the concentration of FAAs over the growing season and also during the warmer sessions due to the high underground completion for nitrogen among the plant roots and microorganisms have been reported (Weintraub and Schimel, 2005). Rapid degradation of soil amino acids by SMC has been demonstrated (Jones et al., 2005; Apostel et al., 2013). There is also a possibility for increase in the population SMC capable of utilizing L-canavanine. Although L-canavanine exists in many legume species, its exudation mechanism and its effect on the soil is unknown.

L-Canavanine is known as a natural insecticide. Its analogous structure to L-arginine can interfere with L-arginine metabolism in plants and animals and, therefore, incorporate in *de novo* proteins and result in protein malfunctions (Mitri et al., 2009).

Previously, L-canavanine was found in the young root of the germinating hairy vetch seeds (Kamo et al., 2015). Kamo et al. (2015) also showed that the L-canavanine content in the hairy vetch root declines over several days after seed germination and it converts to “cyanamide,” the major allelochemical of hairy vetch. L-canavanine likely influences the interaction of hairy vetch with surrounding organisms in the soil, including SMC by limiting or stimulating the growth of selected bacteria. Similar to the 20 canonical proteinogenic amino acids, L-canavanine also contribute to the formation of the soil microbial community that is important in the robustness of the soil.

In our study, hairy vetch grown in *in vitro* agar medium also released L-canavanine through the root system. The seed coats and cotyledons were not in direct contact with the agar medium surface due to the suspended seed culture system, and therefore, the L-canavanine found in the agar medium was directly secreted from the roots and accumulated in the medium.

However, we still do not know if soil bacteria can use L-canavanine as a source of nitrogen. Although there are several reports on the effect of amino acids on SMC, such as disassembly of *Bacillus* species (Aliashkevich et al., 2018), the role of combination and/or single amino acid on the formation of the SMC have not been well studied.

In the agricultural system, any external nutrient sources such as added amino acid from plant roots, or composts have a much shorter lifespan in the soil and therefore may have shorter effect on SMC in comparison with the added agrochemicals (Perez et al., 2015).

### Changes in the Soil Bacterial Dynamics Caused by L-Canavanine

To this date, changes in the SMC by a single non-proteinogenic have not been previously observed. It is known that certain proteinogenic amino acid substrate in the soil increases the microbial biomass yield (Vinolas et al., 2001).

It has also been reported that the L-canavanine acts as an inhibitor of bacteria in the seed surroundings of alfalfa (Emmert et al., 1998) to increase the seed chance of survival. Emmert et al. (1998) also observed the selective effect of L-canavanine on different *Bacillus* sp. *in vitro*. L-canavanine has also been found to be incorporated into the antimicrobial protein in insect and reduced their effects (Rosenthal et al., 1989). This malfunction often happens because the arginyltRNA synthetase can discriminate between L-canavanine and L-arginine during the protein synthesis (Bence and Crooks, 2003).

Alpha diversity measures, such as Faith’s PD index revealed that SMC diversity was significantly lower in applied 100 and 500 nmol/g soil concentrations than control. Increased soil L-canavanine content may reduce the presence of microbial taxa with low abundance in each sample. This could mean L-canavanine may only be consumed by specific taxa, and it may be an inhibitor for the others. Meanwhile, results showed that dissimilarities of the bacterial community increased with an increase in used concentration of the L-canavanine, indicating that the difference of communities between the control and 500 nmol/g soil L-canavanine in the soil increases with the increase in L-canavanine exudation.

Based on their phylogenetic coverage, it appears that Acidobacteria and Proteobacteria phyla are metabolically diverse. Similarly, it is clear that detailed and functional descriptions of Acidobacteria in the soil are important (Kielak et al., 2016). Proteobacteria are capable of transporting a diverse range of substrates including amino acids, carbohydrate, peptides, siderophores, cation, and anions (Rawat et al., 2012; Kielak et al., 2016). The α- and β-Proteobacteria are known to be highly capable of the catabolizing D- and L-amino acids as the source of carbon and nitrogen in the soil (Radkov et al., 2016).

Actinobacteria are involved in the important soil ecosystem cycles, such as carbon, nitrogen, phosphorus, and several other nutrients cycling in the soil (Hill et al., 2011; Zhang et al., 2019). It is not clear why L-canavanine increased the population of the Actinobacteria, but it can be speculated that L-canavanine may increase some Actinobacteria and, as a result, regulate the production of secondary metabolites (enzymatic inhibition during the carbon, nitrogen pathways) (Hill et al., 2011) and loss of nutrients in the early stages of hairy vetch growth.

Bacillaceae family is one of the most abundant microorganisms in the soil and plays an important role in many biological phenomena. They confer health benefits to crop plants and therefore are known as plant growth-promoting rhizobacteria (PGPR) (Fan et al., 2018; Saxena et al., 2020). It can be speculated that hairy vetch might also release L-canavanine at the beginning of seedlings’ lifecycle in order to increase or control the number of PGPR such as *Bacillus* to the benefit of the seedlings, which also might be related to the selective role of L-canavanine in inhibition or promotion of bacteria. Similarly, several bacteria taxa and families decreased or increased in lower magnitudes by application of L-canavanine, which also might be due to the same phenomenon.

### PICRUSt2 and Prediction of Metagenome Functions From 16S Amplicon Data

The reason behind why bacteria uptake and at the same time utilize the nutrient (i.e., amino acids) in complex rich soil ecosystems is still unknown. Yet, it is known that bacteria can change their role in resolving regulatory decisions for uptake and catabolism of alternative carbon sources by inhibition of phosphotransferase system (PTS), and catabolism of alternative carbon sources when they have excess uptake of certain amino acids (Zampieri et al., 2019).

These are a continuous chain pathway related to the superpathway of ornithine that participates in the conversion of the amino acids L-arginine and L-ornithine to the polyamine putrescine in organisms. These will then subsequently be degraded to 4-aminobutanoate, and then convert to 4-aminobutanoate and finally succinate. The succinate is fed into the TCA cycle and energy cycle (Kashiwagi et al., 1991; Kurihara et al., 2005). It might be possible that because L-canavanine is structurally analogous to L-arginine, it might be affected by the similar soil metabolite degradation pathway.

Similarly, the results of this study also showed that L-canavanine changes the catabolism pathways of carbon sources in the soil total metabolite pathway (Figure 7). This might be the case for the hairy vetch strategy by releasing L-canavanine from the root and therefore inhibition of the SMC from consuming the carbon sources (i.e., glucose oxidative degradation pathway). Changes in the glyoxylate cycle in this study due to L-canavanine might help bacteria in energy production and biosynthesis of carbon structure, and therefore, depletion of these pathways might limit the growth of some competing species (Berg et al., 2002) that may benefit the hairy vetch seedlings.

## Conclusion

Our results again highlighted the potential allelopathic effect of the hairy vetch, but this time through its root exudates. It is clear from the outcome of the present study that the exudation of non-proteinogenic amino acid L-canavanine from the root of hairy vetch occurs in the natural field condition. The soil microbial diversity was affected by the L-canavanine as well as the specific microbial populations. We also predict that root exudated L-canavanine can influence the kinetics and patterns of depletions of hydrocarbon degradation and amino acid such as arginine and ornithine degradation pathway. Thus, the underlying mechanism of L-canavanine exudation from the root and in which soil microorganism being affected by L-canavanine in rhizosphere soils needs more attention and is of significant interest for predicting the soil robustness, and organic culture practices.

## Materials and Methods

### Plant Materials and Farm Experimental Design

#### Pot Experiment

Seeds of hairy vetch (*V. villosa*, Roth subsp. *villosa*) were purchased from Takii, Japan, and were grown in a plastic container (20 cm × 20 cm × 60 cm) holding 20 L of commercial soil (Kumiai Nippi Engei Baido No. 1, Zen-Noh Co., Tokyo, Japan) and then were allowed to grow in the greenhouse. After plants reached 20-cm height (3 weeks after germination), 10 plants were gently removed from the pots and placed on a clean plastic sheet. Roots were shaken vigorously to remove any excess soil or organic material. To collect the Rhizosphere soil (Rh), soil adhered to the root were gently brushed off on a clean paper using a clean brush (Daiso Co., Japan). The collected soils were then passed through a 2 mm × 2-mm mesh to remove any excess material and gravels. Bulk soil was collected from plots without cultivation of hairy vetch.

#### Farm Experiment

For the field experiment, hairy vetch seeds were planted in the field (Tokyo University of Agriculture and Technology, TUAT) from November 2017 to February 2018 and were allowed to grow until the upper part of the plant reach 20 cm (vegetative stage). The soil type in the TUAT field was andosol, and no irrigation system was used during the experiment. Ten healthy plants were dug out using a shovel and placed in a plastic bag in a cool box containing dry ice. The rhizosphere soil was collected as described above after transporting the plants to the laboratory. Bulk soil was collected from the plots with no hairy vetch cultivation. Mineral contents in the farm soil were measured using a set of small-scale protocols for analyzing the nutrient minerals of small soil samples as described previously (Yamazaki et al., 2019; Supplementary Table 1).

#### Agar Medium Experiment for Validation of Root Exudation of L-Canavanine

To validate that L-canavanine is released from the root of the hairy vetch and not leached from the seed coat or seed itself, we designed a validation step. In this step, some seeds were surface sterilized using sodium hypochlorite (0.1%, 10 min) and washed with distilled autoclaved water (3-times, 5 min). Ten sterilized seeds were then placed on the top of sterile stands made of aluminum foils to keep 0.5 cm distance from the medium surface in 500 ml beakers containing 60 ml agar medium (pH 8.5). This step was to make sure exudation will not occur from the seed coat. After about 3 weeks, all plants were harvested, and the root and shoots were separated and kept in −80°C for the extraction of amino acids. Moreover, the agar medium was extracted using 80% ethanol immediately for the amino acid analysis step.

### Amino Acid Extraction

Free amino acids were extracted based on the previously established method (Tsuchiya et al., 2013). The amount of 1 g of soil was extracted three times with 80% (v/v) aqueous ethanol (pH adjusted to 3.0 with HCl) at 60°C. This step was to remove most of the extractable amino acid, which is easily available for plant root and other organisms. Combined fractions were evaporated to dryness using a vacuum centrifugal evaporator (CVE-3100; EYELA, Tokyo, Japan), equipped with a cold glass trap (Uni trap UT-1000; EYELA).

### Gas Chromatography-Mass Spectroscopy Analysis of L-Canavanine

For gas chromatography-mass spectrometry (GC-MS), the residues were dissolved in low pH pure water (pH 3.0 adjusted with HCl). To derivatized the amino acids, EZ:Faast GC-MS kit (Phenomenex Inc., Torrence, CA, United States) was used and the standard L-canavanine (M9P5344; Nacalai tesque, Kyoto, Japan) with propyl chloroformate, following the original protocol provided in the kit. The GC-MS system (Shimadzu QP2010) supplied with helium as carrier gas (He, purity 99.999%, Tanaka, Japan) analysis was performed using a gas chromatograph coupled to a mass spectrometer. The oven temperature program was designated as follows: initial temperature 110°C raised to a final temperature of 320°C at 30°C min^–1^. The injection port temperature was adjusted to 250°C. The temperatures of the ion source were 240°C, quadrupole 180°C, and auxiliary at 310°C. A 1.5 μl sample was injected in split mode (1:15). The flow rate was kept at 1.1 ml min^–1^. The MS scan range was adjusted from 45 to 450 m/z. The data of two to three separately extracted samples were expressed as averages with SD. The outputs of GC-MS and chromatogram are shown in Supplementary Figure 3. Moreover, L-canavanine recovery rate of the pot and farm soil was calculated by adding 500 nmol L-canavanine to 1 g of soil and extracting the mixture. GC-MS analysis results revealed that the recovery rate for pot and farm soil were 42 and 40%, respectively (Supplementary Table 2). Studies have shown the recovery of amino acids from hydroponic set ups used in biology is considered about 70–100% (Persson and Näsholm, 2001; Inoue et al., 2015).

### Soil-L-Canavanine Incubation Experiment

To simulate the effect of canavanine on the soil bacteria, different concentrations of L-canavanine [0 (control), 20 (low), 100 (medium), and 500 (high) nmol] was added to 1 g of the filed soil samples in a 5-ml falcon tube. Four replicants for each treatment was made. Samples were then incubated at 25°C for 15 days. L-canavanine treatment was repeated three times during the incubation period. Soil bacterial DNA extraction was performed at the end of the incubation time.

### Soil DNA Extraction and Bacteria DNA Sequencing

DNeasy PowerSoil Kit (QIAGEN, Hilden, Germany) was used to extract the soil DNA from 250 mg of the soil samples. To crush soil bacterial cells, a milling machine was used. The quantity of the extracted DNA was evaluated by Quantus^TM^ Fluorometer and QuantiFluor^®^ Dyes (Promega Corporation, Madison, CA) and stored at 80°C until use.

Polymerase chain reaction (PCR) amplification of the V4 region of 16S rRNA genes was performed following previously described method (Okutani et al., 2020). The 25-μl reaction mixture contained 1 ng of template DNA, 0.3 μl of KOD FX neo (Toyobo, Osaka, Japan), 12.5 μl of buffer (provided with the polymerase), 5 μl of dNTPs (2 mM), and 0.75 μl of 515F (5′-ACACTCTTTCCCTACACGACGCTCTTCCGATCT-GTGCCAGCMGCCGCGGTAA-3′) and 806R (5′GTGACTGGAGTTCAGACGTGTGCTCTTCCGATCT-GGACTACHVGGGTWTCTAAT-3′) primers. PCR conditions were as follows: initial denaturation at 94°C for 2 min, 22 cycles at 98°C for 10 s, 50°C for 30 s, and 68°C for 30 s. The PCR products were purified using AMPure XP magnetic beads (Beckman-Coulter, Indianapolis, IN, United States). For the amplification of bacterial endophytic DNA, 2.5 pmol μl peptide nucleic acid (PNA, Fasmac Co., Ltd., Kanagawa, Japan) were added to the reaction mixture to avoid the amplification of root mitochondrial.

To attach MiSeq adaptors for both bacterial and fungi sequences, the second round of PCR was performed in a 25 μl reaction mixture containing 2 μl template DNA (purified 1st PCR product), 0.3 μl of KOD FX neo (Toyobo, Osaka, Japan), 12.5 μl of buffer (provided with the polymerase), and 0.75 μl of primers provided with Fasmac Co., Ltd. The second PCR products were purified using AMPure XP magnetic beads and confirmed by electrophoresis on 1.5% agarose gels. The DNA concentration was measured using the Quantus^TM^ Fluorometer and QuantiFluor Dyes^®^ (Promega Corporation, Madison, CA) according to the protocol of the manufacturer. Illumina MiSeq platform (2 × 250 bp) was used for the amplicon sequencing of V4 region that was outsourced to Fasmac Co., Ltd. (Kanagawa, Japan). Sequence data have been deposited in the DDBJ (DNA Data Bank of Japan) Sequence Read Archive under the accession number DRA012221.

The sequences were processed and analyzed using the QIIME2 pipeline (version 2019.10) (Bolyen et al., 2019). Raw fastq files were imported into QIIME2, and paired-end sequences were trimmed at first 20 bases, truncated at 200 bases from the start, quality filtered, denoised, and merged using DADA2 (Callahan et al., 2016) with the q2-dada2 plugin in QIIME2. Multiple alignments of the representative sequences were performed using the MAFFT program, and a phylogenetic tree was generated with the FastTree program in the q2-phylogeny plugin (Price et al., 2009; Katoh and Standley, 2013). Taxonomy was assigned to the sequences using the q2-feature-classifier plugin and a Naive Bayes classifier, which was pre-trained on operational taxonomic units (99% identity) from 515F/806R region of sequences on the SILVA rRNA gene database release 132 (Quast et al., 2013; Bokulich et al., 2018). After the DADA2 process, 98,696–198,196 reads per sample were obtained, representing 10,310 ASVs, and they were normalized by rarefaction to 98,000 reads for diversity analysis (Supplementary Figure 4). Alpha and beta diversity analysis was performed using the core-metrics-phylogenetic pipeline in the q2-diversity plugin within QIIME2 to calculate Shannon’s diversity index, observed ASVs, Faith’s phylogenetic diversity, and Evenness for alpha diversity. It also computes Jaccard, Bray–Curtis, and weighted and unweighted UniFrac distances for beta diversity, and generates PCoA plots for each beta diversity metrics. Association between categorical metadata groups and alpha diversity metrics were tested by Kruskal–Wallis and corrected by the Benjamini–Hochberg method, and that for beta diversity were analyzed by PERMANOVA using the R software packages stats and vegan (Oksanen et al., 2015). Differential abundant test for bacterial taxa at the family level was performed using Welch’s *t*-test corrected by the Benjamini–Hochberg method with an FDR cutoff of 0.05. To identify differential abundant metabolic pathways, we used the R software package ALDEx2 (Fernandes et al., 2013, 2014) with an FDR cutoff of 0.05.

### Data Visualization

Figures in the current work are drawn using the R packages gplots (Warnes et al., 2020) and beeswarm (Eklund, 2016), and statistical analyses were done using R software.

## Data Availability Statement

The original contributions presented in the study are included in the article/Supplementary Material, further inquiries can be directed to the corresponding authors.

## Author Contributions

HM-K, SY, MN, TM, YF, RK, AS, and NO-O conceived and designed the experiments. HM-K, MK, MN, and YO performed the experiments. SY, HM-K, and YA analyzed the data. HM-K, SY, MN, YF, RK, AS, NO-O, and YO wrote the manuscript with input from all authors.

## Conflict of Interest

The authors declare that the research was conducted in the absence of any commercial or financial relationships that could be construed as a potential conflict of interest.

## Publisher’s Note

All claims expressed in this article are solely those of the authors and do not necessarily represent those of their affiliated organizations, or those of the publisher, the editors and the reviewers. Any product that may be evaluated in this article, or claim that may be made by its manufacturer, is not guaranteed or endorsed by the publisher.
