# Supplementary material for: L-Canavanine, a Root Exudate From Hairy Vetch (Vicia villosa) Drastically Affecting the Soil Microbial Community and Metabolite Pathways
Source: Front Microbiol. 2021 Sep 27;12:701796. doi: 10.3389/fmicb.2021.701796 (PMC8503639; doi:10.3389/fmicb.2021.701796)
Supplement: Supplementary file 1 [file Table_1.DOCX]

| Soil parameters | Commercial soil (pot soil)  (Added nutrients) | Farm soil (collected on July 4th, 2018)  (Extracted nutrients) |
| --- | --- | --- |
| Total inorganic nitrogen | 240 mg/kg | 78.66 mg/kg |
| Ammonium nitrogen | 192 mg/kg | 5.29 mg/kg |
| Nitrate nitrogen | 48 mg/kg | 73.37 mg/kg |
| Phosphate | 3000 mg/kg | 62.27 mg/kg (Available P) |
| Potassium | 240 mg/kg | 780.24 mg/kg (Exchangeable K) |
| Magnesium | 240 mg/kg | 480.08 mg/kg (Exchangeable Mg) |

Nutrient contents report of the commercial soil (Kumiai nippi engei baido) is reported in the link below:

<https://www.nihonhiryo.co.jp/product/gardening/images/pdf/janippiengeibaido1goL.pdf>

**Method**

***TUAT Farm Soil mineral analysis***

Mineral contents in the farm soil were measured using a set of small-scale protocols for analyzing the nutrient minerals of small soil samples as described previously (Yamazaki et al., 2019). Briefly, inorganic N (NH_4_-N, NO_3_-N) was extracted using a potassium sulfate solution and measured by colorimetric analysis. The available P content was estimated using the Truog method: extraction using a weak acidic solution and measurement by colorimetric analysis. Exchangeable bases (K, Mg) were extracted using an ammonium acetate solution and measured by flame photometry for K and by atomic absorption spectrometry (AA-6200; Shimadzu, Kyoto, Japan) for Mg.

**Reference**

﻿Shinichi Yamazaki, Kumiko Ochiai, Junko Motokawa, Shoichiro Hamamoto, Akifumi Sugiyama, Masaru Kobayashi. Properties of rhizosphere soil associated with herbaceous plant roots analyzed using small-scale protocols. *bioRxiv* 800664 (2019) doi:10.1101/800664.
