# Supplementary material for: L-Canavanine, a Root Exudate From Hairy Vetch (Vicia villosa) Drastically Affecting the Soil Microbial Community and Metabolite Pathways
Source: Front Microbiol. 2021 Sep 27;12:701796. doi: 10.3389/fmicb.2021.701796 (PMC8503639; doi:10.3389/fmicb.2021.701796)

Standard L-canavanine GCMS derivative output

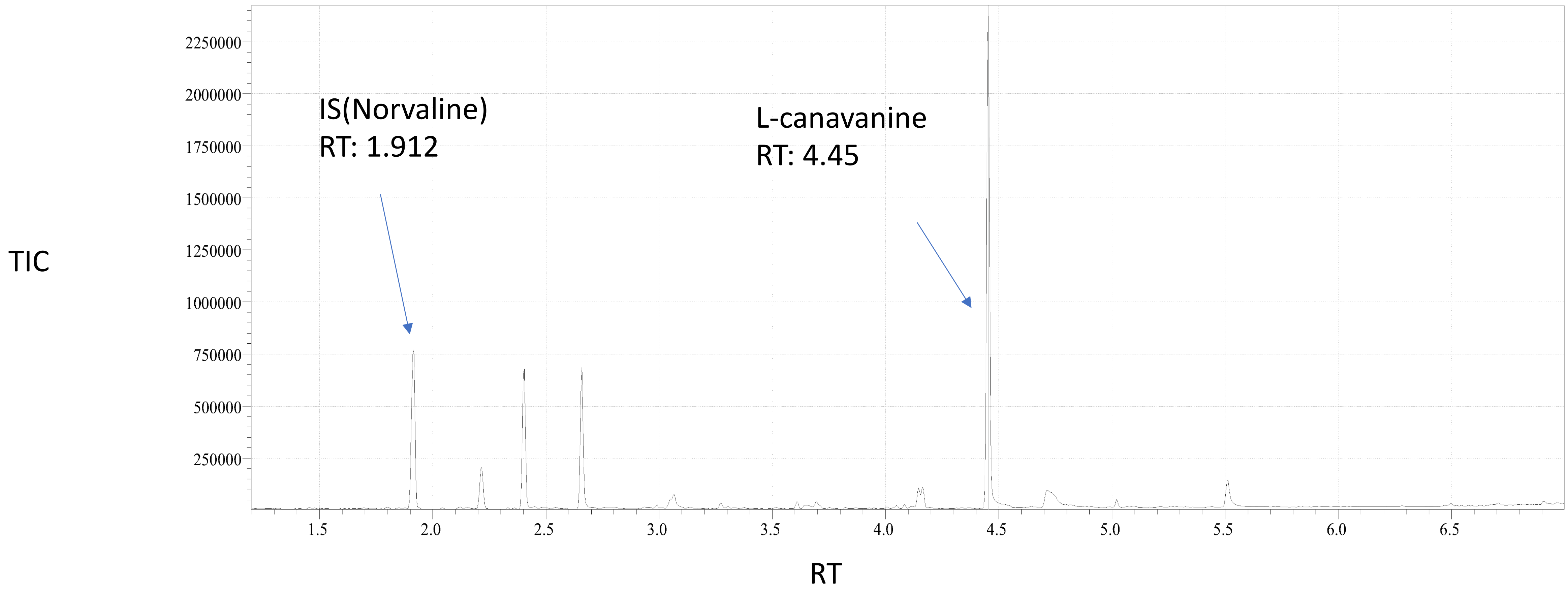

Agar_ hairy vetch exudate _L-canavanine
GCMS derivative output

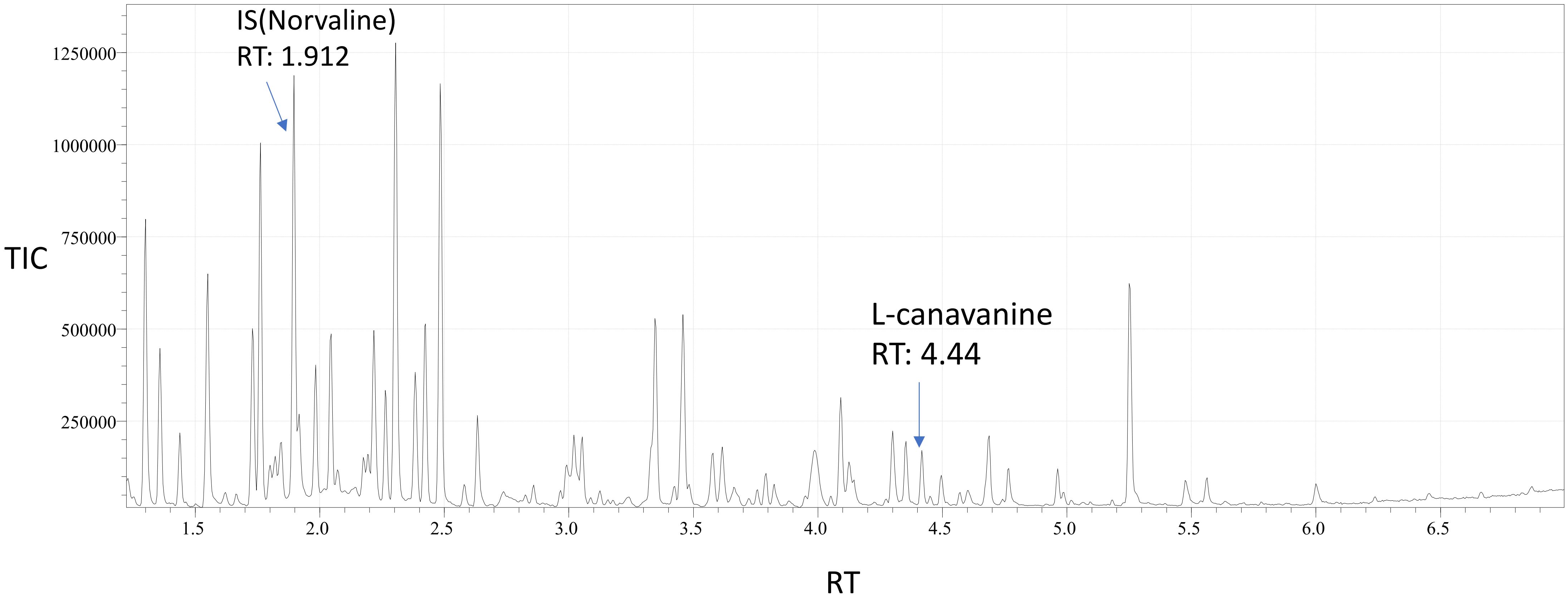

Pot soil_ hairy vetch rhizosphere _L-canavanine
GCMS derivative output

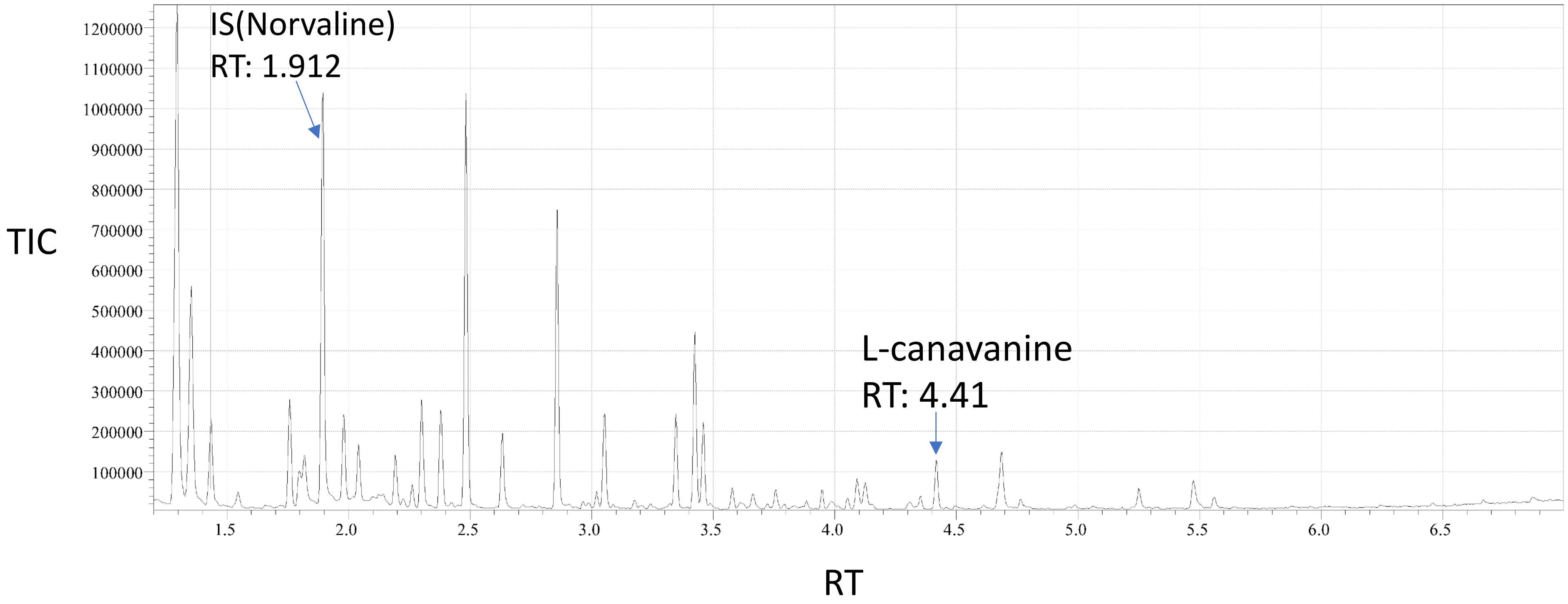

Farm soil _ hairy vetch rhizosphere _ L-canavanine
GCMS derivative output

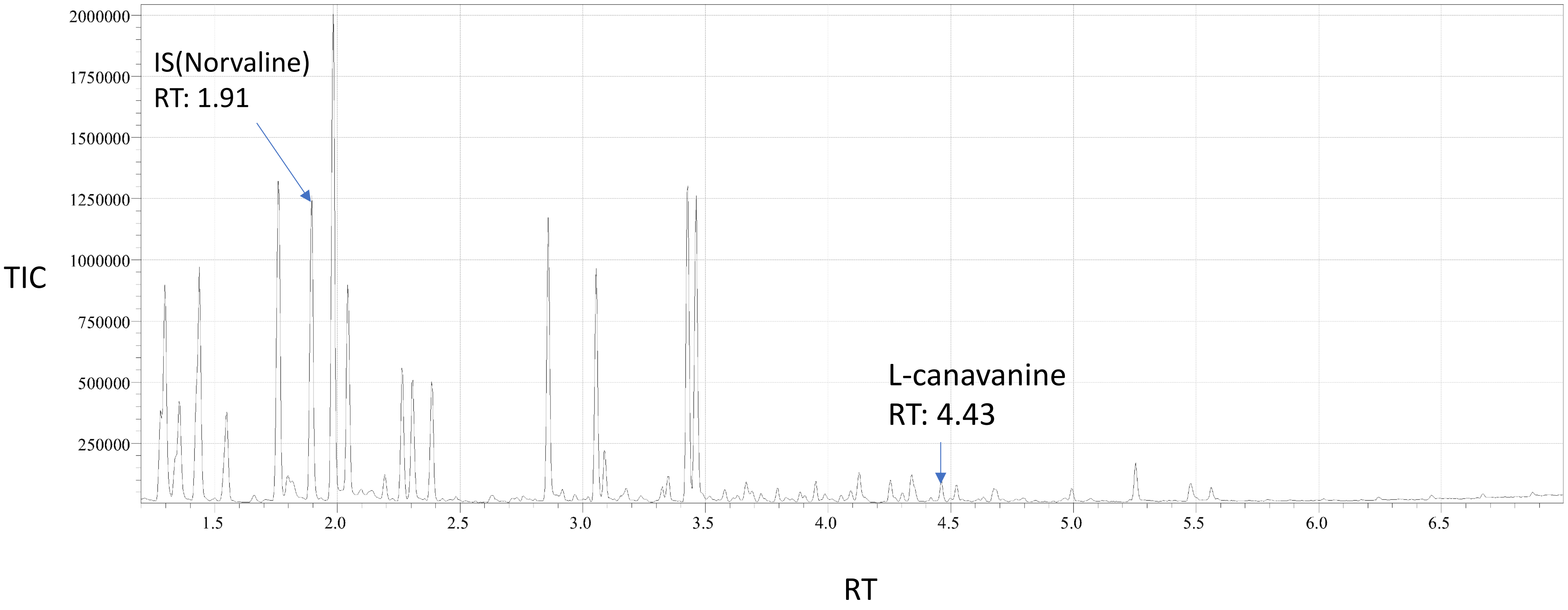

Standard L-canavanine GCMS derivative mass pattern verses target pattern

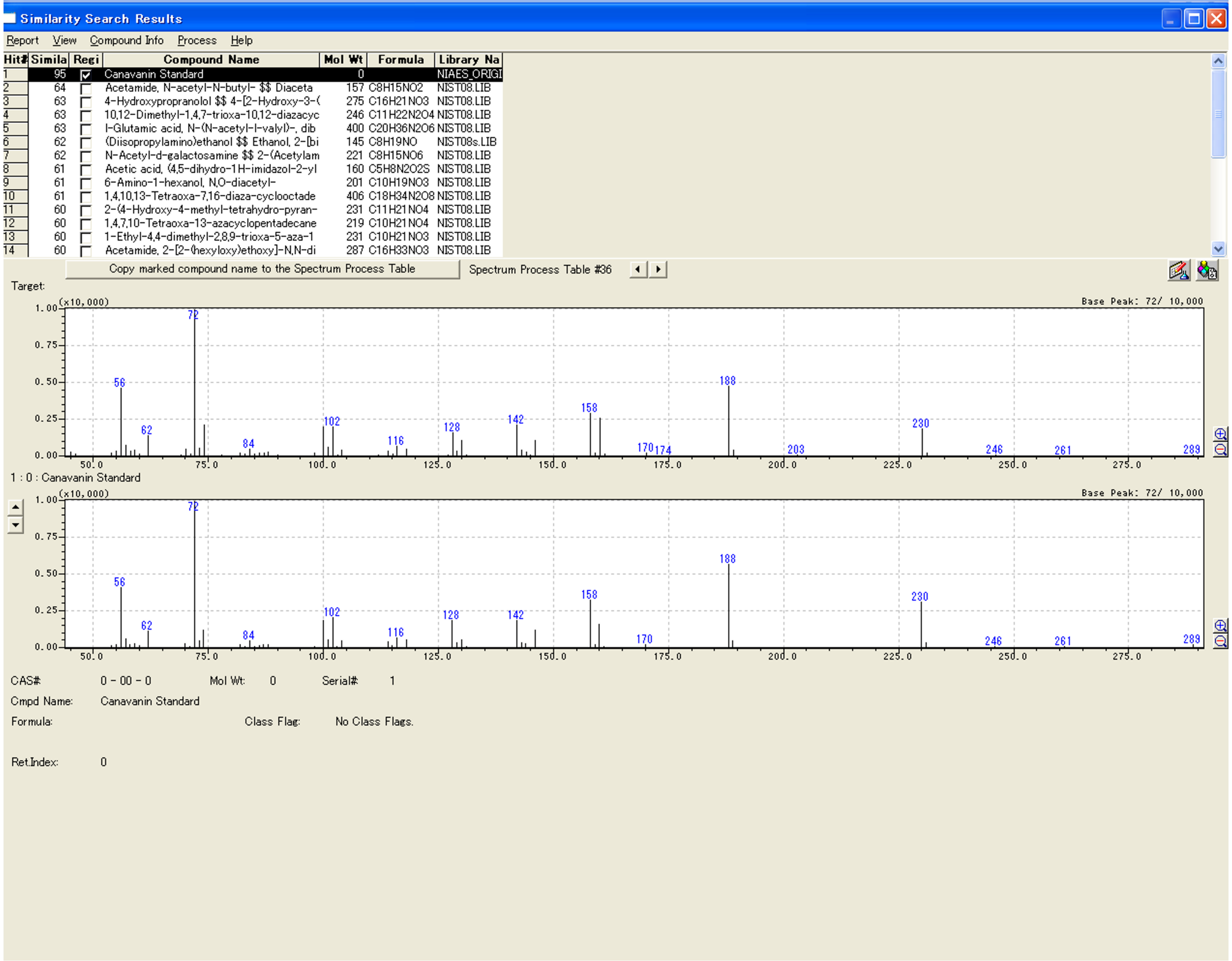

Supplement: Supplementary file 3 [file Data_Sheet_1.PDF]
