# Supplementary figures and images for: L-Canavanine, a Root Exudate From Hairy Vetch (Vicia villosa) Drastically Affecting the Soil Microbial Community and Metabolite Pathways
Source: Front Microbiol. 2021 Sep 27;12:701796. doi: 10.3389/fmicb.2021.701796 (PMC8503639; doi:10.3389/fmicb.2021.701796)

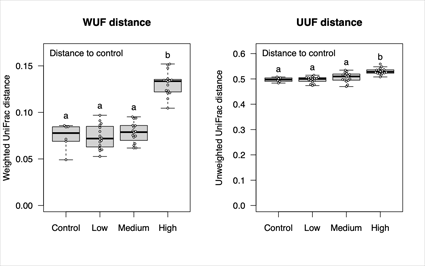

Supplement: Supplementary file 4 [file Image_1.TIFF]

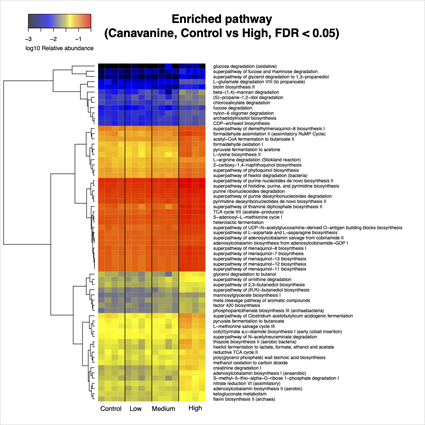

Supplement: Supplementary file 5 [file Image_2.TIFF]

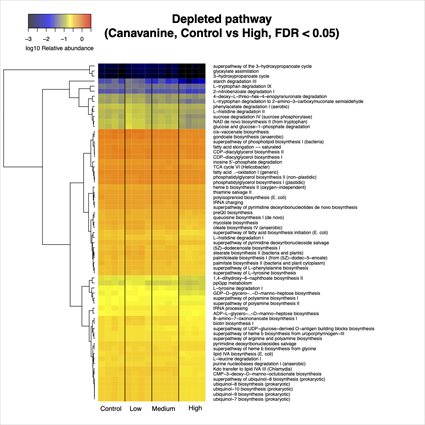

Supplement: Supplementary file 6 [file Image_3.TIFF]

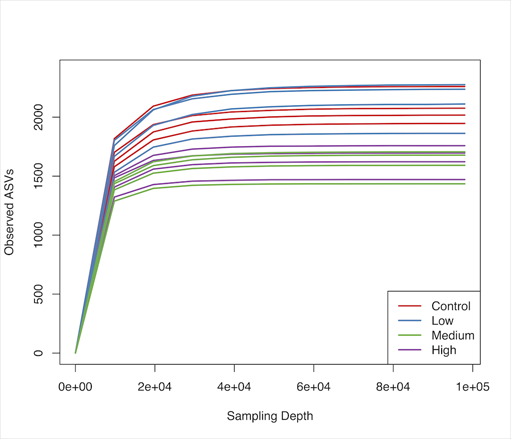

Supplement: Supplementary file 7 [file Image_4.TIFF]
